# Multi-Target PIR Indoor Localization and Tracking System with Artificial Intelligence

**DOI:** 10.3390/s22239450

**Published:** 2022-12-02

**Authors:** Xuan-Ying Chen, Chih-Yu Wen, William A. Sethares

**Affiliations:** 1 Department of Electrical Engineering, National Chung Hsing University, Taichung 40227, Taiwan; 2 Department of Electrical Engineering, Bachelor Program of Electrical Engineering and Computer Science, Innovation and Development Center of Sustainable Agriculture (IDCSA), National Chung Hsing University, Taichung 40227, Taiwan; 3 Department of Electrical and Computer Engineering, University of Wisconsin-Madison, Madison, WI 53706, USA

**Keywords:** pyroelectric infrared sensors, non-wearable system, multiple targets localization, artificial intelligence, deep learning, data augmentation strategy

## Abstract

Pyroelectric infrared (PIR) sensors are low-cost, low-power, and highly reliable sensors that have been widely used in smart environments. Indoor localization systems may be wearable or non-wearable, where the latter are also known as device-free localization systems. Since binary PIR sensors detect only the presence of a subject’s motion in their field of view (FOV) without other information about the actual location, information from overlapping FOVs of multiple sensors can be useful for localization. This study introduces the PIRILS (pyroelectric infrared indoor localization system), in which the sensing signal processing algorithms are augmented by deep learning algorithms that are designed based on the operational characteristics of the PIR sensor. Expanding to the detection of multiple targets, the PIRILS develops a quantized scheme that exploits the behavior of an artificial neural network (ANN) model to demonstrate localization performance in tracking multiple targets. To further improve the localization performance, the PIRILS incorporates a data augmentation strategy that enhances the training data diversity of the target’s motion. Experimental results indicate system stability, improved positioning accuracy, and expanded applicability, thus providing an improved indoor multi-target localization framework.

## 1. Introduction

In recent years, indoor localization has become a key area for Internet of Things (IoT) applications, especially in a mall, a hospital, or a big office environment. Although several approaches have been proposed for localization applications (e.g., systems with a camera, wearable devices, or ultrasonic sensors), pyroelectric infrared (PIR) sensors provide a useful trade-off between privacy and estimation accuracy in indoor localization systems. Since a binary PIR sensor detects only the presence of a human motion in its field of view (FOV) without location information, utilizing the information of the overlapping FOVs of multiple sensors can be useful for localization. The operational principle of a PIR sensor is to interpret the thermal variations caused by target (human) motions occurring in its FOV. 

Considering the practicalities, instead of using a wall-mounted deployment or a floor-deployed scheme, this work investigates the tracking performance with a ceiling-deployed strategy. Although PIR sensor applications can be found in the literature, focusing on human activity recognition, monitoring, and tracking, the feasible PIR implementations are modest. Moreover, although the existing studies combine the properties of PIR sensors and spatial information to estimate the target position with the help of deep learning algorithms, most of them lack an analysis concerning the impact of the behavior of the learning model on accuracy performance and implementation complexity. Hence, the challenge of performance improvement (e.g., explaining the behavior of the learning model, dealing with data processing, increasing response speed, and suppressing estimation error) of the PIR sensor network remains an important problem.

Several existing schemes using PIR-based localization show the potential of incorporating a deep learning component: modular learning in [1], a permutation-invariant approach to signal separation in [2], and target occupancy detection in [3]. Modular learning decomposes a problem into several sub-tasks and then utilizes multiple neural networks to solve them separately. However, the method is limited due to the indeterminate number of sub-tasks (i.e., generating n detection modules in n-target scenarios). A permutation-invariant approach to signal separation uses permutation-invariant training (PIT) to address the label permutation problem at training time in the cocktail party problem using information from reference signals. A limitation of this approach is that it is difficult to know the reference signals in advance, especially for mixed signals in real environments. Instead, this work integrates the permutation invariance scheme with the changing tendency of the label score (see Section 3.4) to determine possible target positions.

The contributions and key features of this paper are to show the usability of the proposed data preprocessing method (i.e., the Shuffle Sampling (SS) scheme, detailed in Section 3.1) and PIR sensor analog outputs in recognizing multi-target movement with the help of deep learning algorithms (i.e., the LSTM model in combination with the permutation invariant strategy of Section 3.4) and the development of an approach to explain the behavior of the ANN model. Thus, this paper extends our previous work in [3] to the PIRILS, which provides for the simultaneous localization of multiple targets by using an ANN. Specifically, the PIRILS constructs a monitor window of the multi-target locations that relates the PIR sensor’s alarm sequences to target locations. To further improve the performance of the ANN model, the PIRILS incorporates a data augmentation strategy based on the characteristics of the monitoring window. With the help of deep learning algorithms, this helps explain the behavior of the ANN model and the quantization of the position estimation.

The remainder of this paper is organized as follows: Section 2 describes related work about PIR sensing and localization methods. Section 3 extends our preliminary design concept in [4] and demonstrates the data augmentation strategy and deep learning techniques that form the heart of the PIRILS. Section 4 reports the experimental results and evaluates the system’s performance. Discussions are presented in Section 5. Conclusions are made for further research in Section 6.

## 2. Related Works

The state-of-the-art in PIR-based localization with an artificial neural network (ANN) can be found in [1,3,5,6]. Yang et al. [1] proposed the PIRNet architecture to localize the target, where the ANN has been composed by the target counting network and an M-person localization network. Yang et al. [5] then extend their method to predict information about the targets’ location relative to each PIR sensor. Their model, DeepPIRATES, further infers the targets’ absolute locations using a particle filter that integrates information about the relative locations of the targets and the absolute locations of the PIR sensors. Yun et al. [6] proposed a deep learning method that combines a convolutional neural network (CNN) with the PIR sensor analog outputs to recognize the direction of human movements (e.g., capturing the analog output signals of the PIR sensors while the targets move throughout the eight directions of a monitored area). Andrews et al. [3] propose a usability method of PIR sensor for stationary human presence detection and biometric authentication based on the chest motion of human subjects using a Long Short-Term Memory (LSTM) model, where the experimental result indicates that biometric authentication using PIR sensor data has a strong dependence on temporal information.

**Figure 1:**
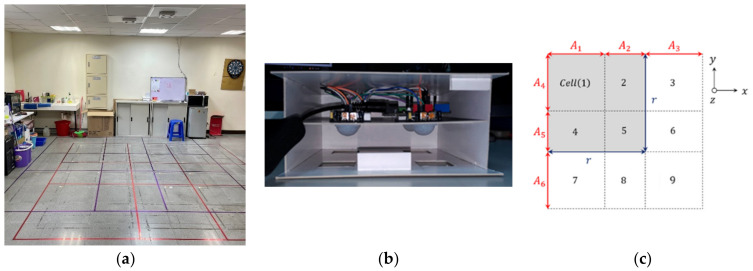
(**a**) The experimental scenario; (**b**) the electronic equipment of our work; (**c**) the detection of target movement. We refer the reader to [4] for more details.

Table 1 summarizes the characteristics of several PIR detection systems in terms of the sensor location, signal preprocessing techniques, and observation space. As shown in Figure 1 and referring to our previous work [4], this paper extends its easy-to-implement system structure to the PIRILS, which includes the sensing signal processing and the usability method of the existing system for the multi-target detection using an ANN model. In the experimental settings, we explore the impact of signal-processing algorithms and the constructs of the ANN model on estimation accuracy and make a comparison among the proposed refinement approaches (as detailed in Section 3 and Section 4).

## 3. PIRILS

This section briefly describes the proposed multi-target tracking system, including data collection procedures, data preprocessing schemes, and ANN architecture. As currently implemented, the PIRILS assumes that it knows the number of targets to be tracked. The system then determines the locations of the targets and tracks their positions over time.

### 3.1. Data Collection

Data are collected at a sampling frequency of 50 Hz over an (approximately) 5 s time interval, leading to a 256-sample window size. Referring to the layout of the test field in Figure 1a [4], data were only collected as the targets entered the sensing field. Thus, the 256-sample window provides a complete description of the signal waveform and sensor response to human movement. All captured data were collected from two subjects, an average-sized male (medium frame of 5′6″ with a weight of 145 pounds) and an average-sized female (medium frame of 5′3″ with a weight of 132 pounds). Data were recorded in a real-time manner from a Bluno [8], the embedded development board, and then transferred to a laptop via a universal serial bus (USB) cable, as shown in Figure 1b [4]. The analog voltage output of the PIR sensor signal was quantized from the range of 0 to 5 V to a range of 0 to 1023 digital states using the ADC input pin of the Bluno.

Labels for locating zones were added manually. Integer labels are given for each zone parameter. Figure 1c [4] shows the labels for possible locations of target movement. Accordingly, the ANN is trained to learn the layout by matching the labels. Figure 1c shows the reference structure used to divide the detection area of four PIR modules into nine cells, where the (square) detection area of a PIR module (gray area) covers four cells (say Cells 1, 2, 4, and 5) with side length $r=A_{1}+A_{2}=A_{4}+A_{5}$, where $A_{1}=A_{3}=A_{4}=A_{6}=1.23\;m$ and $A_{2}=A_{5}=0.87\;m$. Table 2 shows that each cell is covered by the detection area of the PIR module in the detector, where 1 (resp.0) means that the detection area of the PIR module covers (resp. does not cover) this cell.

### 3.2. Data Preprocessing

The short-time energy (STE) method is applied to normalize the analog PIR voltage signals such that the target motion and the state of each PIR sensor can be described clearly [1,9]. With a single PIR detector, a C×P matrix, $Cov_{det}$, for the detection area by each cell covered is represented as

(1) $$\begin{array}{cc} Cov_{det}={\left[cov_{c}\right]}_{C\times 1}, & \end{array}$$

where c is the cell index, C is the number of cells in the detection area of a detector. $cov_{c}=\left[\begin{array}{ccc} cov_{c,1} & \ldots & cov_{c,P} \end{array}\right]$ is a 1×P row vector. $cov_{c,p}$ is one if the pth PIR sensor is covered and is zero if uncovered.

**Figure 2:**
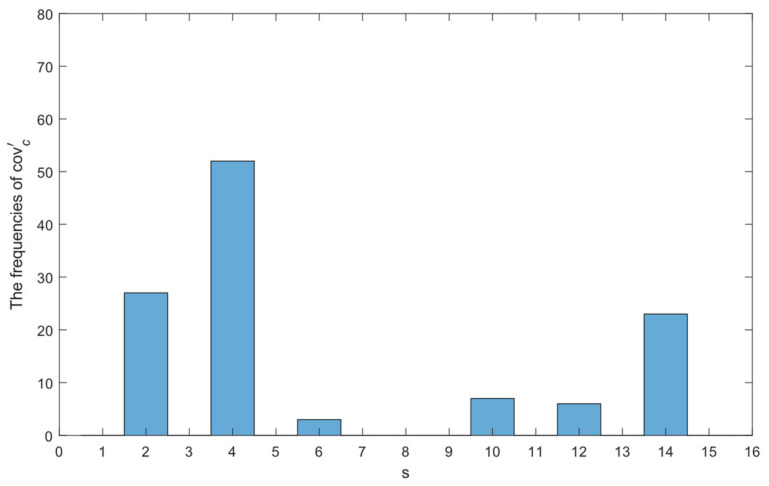
The PIR statistical distribution of two targets detected in Cell (3) and Cell (8) within a time frame.

The elements of $Cov_{det}$ are encoded in a P-bit format, which yields:

(2) $$cov_{c}^{\prime }=\overset{P}{\underset{p=1}{\sum }}cov_{c,p}\times 2^{p},$$

and counts the frequencies of all the $cov_{c}^{\prime }$ in a window of 256 samples. Figure 2 shows an example, illustrating the statistical distribution of the proposed system as two subjects are moving in the detection cells. Note that s represents the value of $cov_{c}^{\prime }$, which is the binary to decimal conversion based on actual target presence (ranging from 0 to 15), as demonstrated in Table 2.

Taking a PIR detector with P=4 and C=9 in this work, $Cov_{det}$ can be represented as follows:

(3) $$\;\;\;\;\;\;Cov_{det}={\left[\begin{array}{ccc} cov_{1,1} & \cdots & cov_{1,4}\\ \vdots & \ddots & \vdots \\ cov_{9,1} & \cdots & cov_{9,4} \end{array}\right]}_{9\times 4}={\left[cov_{c}\right]}_{9\times 1}=\left[\begin{array}{c} \left[\begin{array}{cccc} 1 & 0 & 0 & 0 \end{array}\right]\\ \vdots \\ \left[\begin{array}{cccc} 0 & 0 & 0 & 1 \end{array}\right] \end{array}\right]\;$$

In this work, machine learning methods are trained by providing appropriately labeled data, which suggests that the success of the classification with a manual data set depends on reliable data labeling. Selecting an appropriate representation of the input data can be achieved by incorporating domain knowledge, that is, by using prior knowledge of the character of the PIR sensors and the PIR detector [6]. For the PIR target tracking problem, PIRILs use the SS scheme, an oversampling technique that aims to mitigate the overfitting problem due to a small dataset and to increase the variance of the dataset so that data diversity can be achieved. Specifically, SS improves the testing performance by simulating additional training data based on our prior knowledge of the task to be solved. Ideally, the ANN model utilized for PIR-based localization should perform well regardless of the target motion (e.g., speed, pace, and arm motion, etc.). 

**Figure 3:**
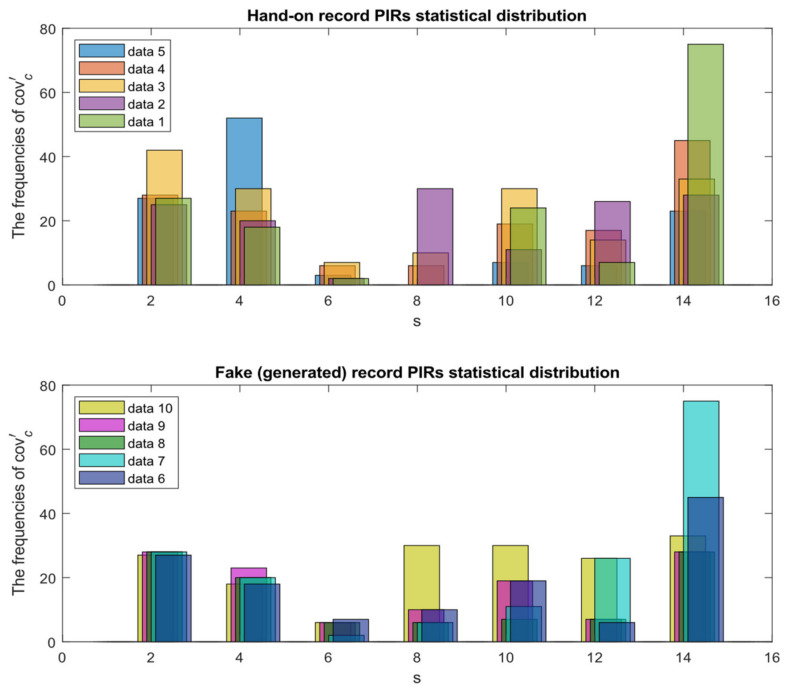
Comparison of the hand-on data (**top**) and the generated data (**bottom**).

Building such an ANN model requires abundant training data to span many different experimental situations. One approach is to use a generative adversarial network (GAN), as in [10], to augment the number of training samples. In the SS method, there are five data records in a label set. First, we shuffle the queue of the data records. Then, we generate a new data record for each $cov_{c}^{\prime }$ by 5 combinations of 1. Figure 3 compares the hands-on data with the generated data, which clearly shows that the method increases the amount of data, where the SS method augments the $cov_{8}^{\prime }$ data from three samples (data 2, data 3, and data 4) with the addition of five newly generated samples (data 6, data 7, data 8, data 9, and data 10). Section 4.3 describes the training accuracy improvement of the ANN model with the SS method.

### 3.3. Artificial Neural Network Architecture

**Figure 4:**
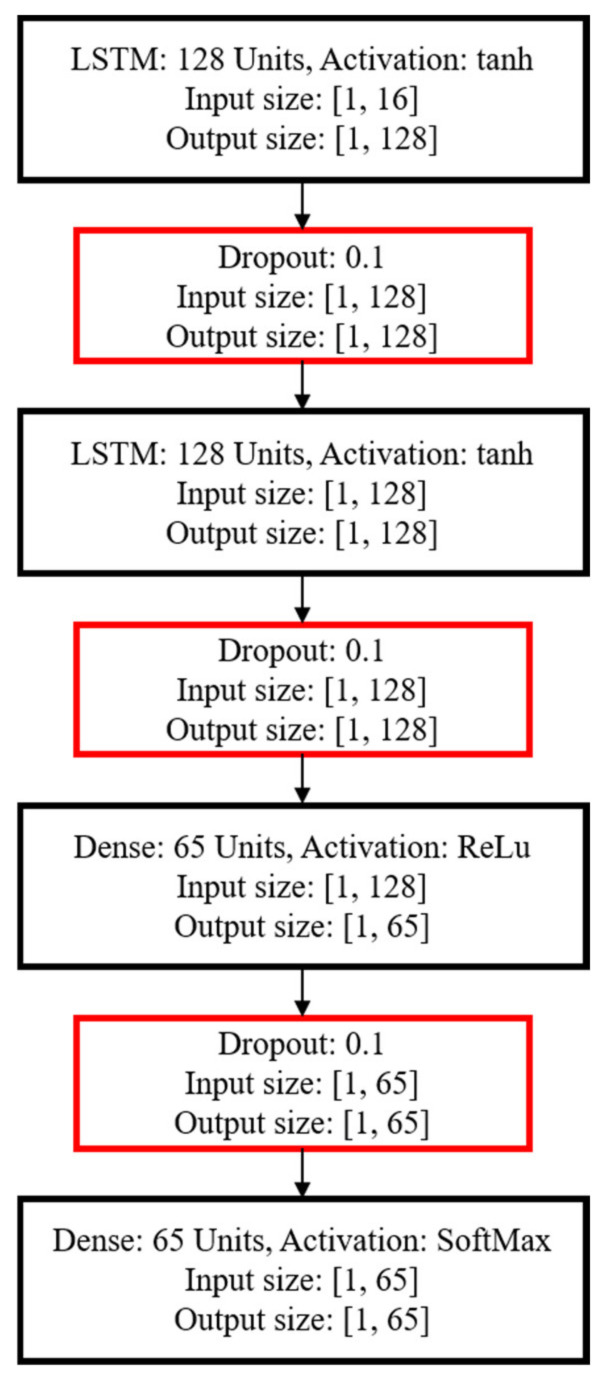
ANN architecture for multi-target tracking, featuring two LSTMs and two dense layers.

We chose the deep learning architecture based on the results in [2] as a basis for the development of an ANN for the multi-target tracking system PIRILS. The ANN input tensor is composed of the $cov_{c}^{\prime }$ statistic in each frame (as in Section 3.2). The architecture is shown in Figure 4. The feature set is fed into a 128-dimension LSTM unit, and then fed into a dropout layer with a weight of 0.1 to reduce overfitting in the neural network. The activation functions for the LSTM and dense layers are the hyperbolic tangent (tanh) and the rectified linear unit (ReLu), respectively, while the final layer uses SoftMax. Categorical cross entropy and Adam are used for the loss function and optimization of the training step, respectively. Note that the output of the neural network is a vector representing estimated target locations.

### 3.4. Permutation Invariance

Tracking multiple targets can be viewed as a kind of source separation problem. This can be formulated as a problem of permutation invariant labels, as is performed in [2] for the audio separation of multiple speakers. We adopt a similar strategy tailored to the features of PIR sensors. However, it may be difficult to know the reference signals for the targets in advance, especially for mixed signals in real environments. Therefore, without reference signals, this work integrates the permutation invariance scheme and the changing tendency of the label score (detailed in Algorithm 1) to determine possible target positions.

The internal evaluation method of Keras (the Python API used to implement the ANN) is based on the accuracy of classification. Accordingly, the proposed Algorithm 1 provides an interpretation of each estimated target position and a corresponding classification label. Here, the positions of targets are individually divided to evaluate the classification accuracy. We uniformly allocate the score for classification model accuracy. Let $score_{true}^{i}$ be the predicted score of the true position of the target i, where the model’s predicted result conforms to the true label ($solution_{n}^{i}$) of the target i in data n. Moreover, let $score_{deviation}^{i}$ be the model associated with predicting the score of adjacent neighboring positions, which belongs to a set of adjacent neighboring labels of the true label of the target i in data n, $other_{n_{c}}^{i}$.

For example, suppose that two targets are detected, and the event outcome is Cell (4, 3). Following Figure 1c, the output of the ANN model likely has the following false situations:

◼ Item 1: target 1 is detected with an adjacent Cell number (e.g., Cell (1, 3), Cell (7, 3))
◼ Item 2: target 2 is detected with an adjacent Cell number (e.g., Cell (4, 2), Cell (4, 6))
◼ Item 3: The detected Cell numbers are inverted (i.e., Cell (3, 4))
◼ Item 4: target 1 is detected with an inverted Cell number (e.g., Cell (3, 1), Cell (3, 7))
◼ Item 5: target 2 is detected with an inverted Cell number (e.g., Cell (2, 4), Cell (6, 4))

The above situations are considered to outline the behavior of the deep learning model in the PIRILS. As depicted in Algorithm 1, the complete process is sequentially executed. First, the classification performance of each target is evaluated to judge its true position and the adjacent neighboring positions (as in the example above in Items 1 and 2) and explore the changing tendency of the label scores, $score_{true}^{i}$ and $score_{deviation}^{i}$. When the above conditions are incompatible and there is not a positive tendency in the changes of $score_{true}^{i}$ and $score_{deviation}^{i}$, then the permutation invariance scheme is applied to swap the order of the target index (e.g., Cell (i, j) transfer Cell (j, i)) and then determine the inverted position (as in Item 3) and adjacent neighboring positions (as in Item 4 and Item 5), respectively. Finally, the output behavior of the ANN for the target permutation invariant problem is characterized.
**Algorithm 1:** Model Performance EvaluationInput: Denote $cell_{n}^{i}$ as the predicted cell number of the target i in data n.
Output: Denote $score^{i}$ as model evaluation score with the target i.
i: the target index
c: the cell index
n: the index of data samples
K: the cell index set of actual target presence and neighboring cells
$K^{\prime }$: the number of neighboring cells (i.e., $K^{\prime }=K-\left\{i\right\}$)1: ▶ Initialize:2: $score_{true}^{i},\;score_{deviation}^{i},\;score^{i}\leftarrow 0$3: ${score}_{true}\leftarrow \frac{1}{I}$           ▷ Maximum target number I.4: ${score}_{deviation}\leftarrow \frac{1}{2\times I}$      ▷ The evaluation scores.5: n←06: ▶ Start:7: While n < the number of data records for model prediction8: ▶ Initial Sequence (target i in location $cell_{n}^{i}\left(c\right)$)9: for 0<I10:  for $0\;<\;\left|K\right|$ (i.e., the cardinality of the set K)11:        if $cell_{n}^{i}$ is equal to $solution_{n}^{i}$12:        $\;\;score_{true}^{i}\leftarrow score_{true}^{i}+{score}_{true}$13:       else if $cell_{n}^{i}$ is equal to $other_{n_{c}}^{i}$14:  $score_{deviation}^{i}\leftarrow score_{deviation}^{i}+{score}_{deviation}$15:  else
16:  $score_{deviation}^{i}\leftarrow score_{deviation}^{i}$17:  end if18:  end for19:  end for20:  ▶ Permutation Invariant (target i in location $cell_{n}^{j}(c)$)21:  ▷ *c* ∈K and $\in \;K\;\prime \left(=K-\left\{c\right\}\right)$22:  for ∀j∈K′23:  if $score_{true}^{j}$ and $score_{deviation}^{j}$ do not increase24:  for $0<\left|K\right|\;\left(i.e.,\;\mathrm{the}\;\mathrm{cardinality}\;\mathrm{of}\;\mathrm{the}\;\mathrm{set}\;K\right)$25:  if $cell_{n}^{j}$ is equal to $solution_{n}^{j}$26: 
$score_{true}^{j}\leftarrow score_{true}^{j}+{score}_{true}$27:  else if $cell_{n}^{j}$ is equal to $other_{n_{c}}^{j}$28:  
$\;\;\;\;score_{deviation}^{j}\leftarrow score_{deviation}^{j}+{score}_{deviation}$29:  else30:  $score_{deviation}^{j}\leftarrow score_{deviation}^{j}$31:  end if32:  end for33:  end if34:  end for35:  n←n+136:  end while“▶” is the comment of main step and “▷” is the comment of note.

To estimate the overall behavior of the ANN model, we calculate the total score of every label, $score^{i}$ of Algorithm 1,

(4) $$\begin{array}{cc} score^{i}=score_{true}^{i}+score_{deviation}^{i}, & with\;i=1,\ldots ,\;I \end{array}\;$$

and then normalize $score^{i}$ between 0 and 1 (norm length 1). The numerical results are detailed in Section 4.3. 

**Figure 5:**
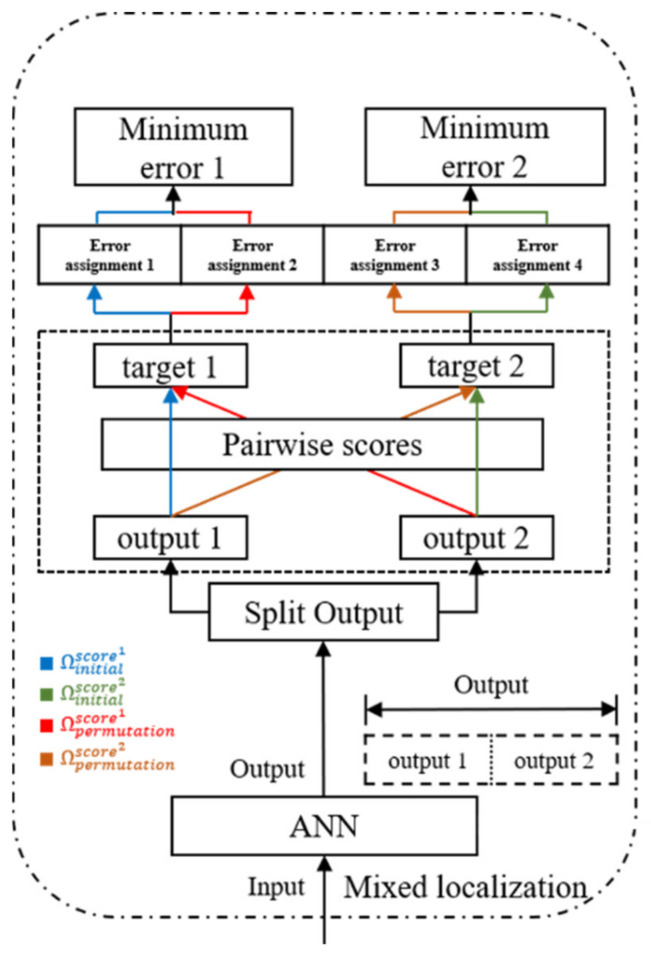
The two-target separation flowchart with permutation invariant evaluation.

To demonstrate the feasibility of this method, consider the two-target scenario. Figure 5 shows the two-target separation flowchart with permutation-invariant evaluation. Denote $cell_{n}^{i}$ as the predicted cell number of the target i in data n. Note that the input to the ANN model is a feature vector with mixed localization. The ANN model outputs an indicator of target presence that is a concatenation of cell numbers (e.g., ($cell_{n}^{1},\;cell_{n}^{2}$)). Then, the output is split to represent the individual targets (e.g., $cell_{n}^{1}$ and $cell_{n}^{2}$). The structure of the pairwise scores borrows the idea of uPIT [2], which is a popular architecture for speech separation, solving the label permutation problem. We associate the reference positions for targets one and two (i.e., ($solution_{n}^{1},\;other_{n_{c}}^{1}$) and ($solution_{n}^{2},\;other_{n_{c}}^{2}$)), to the output locations $cell_{n}^{1}$ and $cell_{n}^{2}$, by computing the pairwise scores between each reference position $\left(solution_{n}^{i},\;other_{n_{c}}^{i}\right)$ and each output location $cell_{n}^{i}$. Thus, we then determine the (total of I!=2!) possible permutations between the references and the output positions,

(5) $$\Omega_{permutation}^{score^{i}}=output\;1\;\;,target\;2,\;i=1$$

(6) $$\Omega_{permutation}^{score^{i}}=output\;2\;\;,target1,\;i=2$$

and compare the positioning performance with those of the initial order,

(7) $$\Omega_{initial}^{score^{i}}=\left\{output\;1,\;target\;1\right\},\;i=1\;$$

(8) $$\Omega_{initial}^{score^{i}}=\left\{output\;2,\;\;target\;2\right\},\;i=2\;$$

where output 1 is $cell_{n}^{1}$, output 2 is $cell_{n}^{2}$, target 1 is ($solution_{n}^{1},\;other_{n_{c}}^{1}$), target 2 is ($solution_{n}^{2},\;other_{n_{c}}^{2}$). Consequently, for the two targets, the predicted cell numbers with the lowest error are chosen, and the locations are optimized to reduce the MAE errors.

### 3.5. Implementation of the Localization System

**Figure 6:**
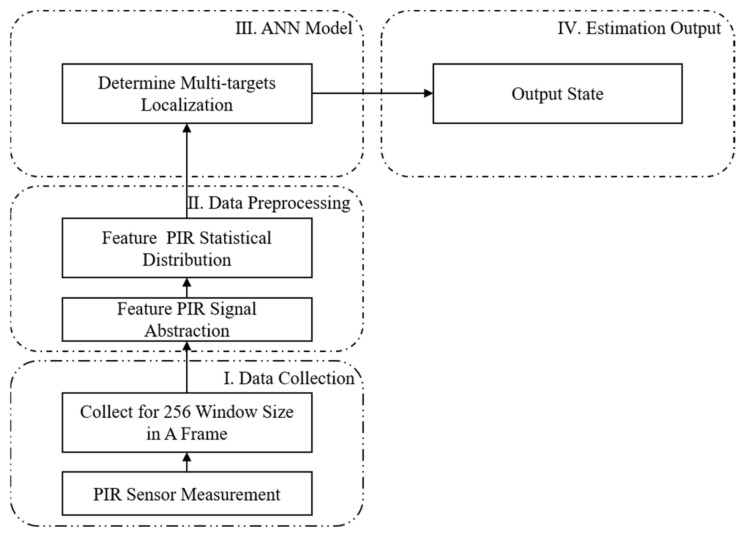
Flow chart of the proposed system for multi-target localization.

Figure 6 shows a flowchart of the proposed solution for multi-target decision schemes, including four parts: data collection (Part I), data preprocessing (Part II), ANN model (Part III), and estimation output (Part IV). Part I collects 256 data samples in a frame from serial PIR sensor measurements. Part II applies the short-time energy scheme to clearly indicate the state of each PIR sensor, and then counts the cell numbers to build a statistical distribution, which is fed into the ANN model. Part III has the ANN models. Section 3.4 describes the behaviors of LSTM model, where the results are shown in Section 3.3. Part IV includes a web-based visualization interface that shows the cartesian coordinates of multiple targets.

## 4. Experimental Results

This section describes the behaviors of the ANN model and further compares the localization errors of the PIRILS with existing PIR-based methods in two-target localization.

### 4.1. Dataset

A dataset of 320 samples is applied to train all models with PIR statistical distributions. Note that considering the signal resolution, the central Cell (5) is excluded in the test trails. Thus, with the cell layout of Figure 1c, the zone classification parameter is labeled from 1 to 64 for the movements of two targets with five data records per label. Referring to the experimental settings in [6], the models give a stable performance with at least ten data records. As a result, each label in this work is constructed with 10 samples by the proposed SS scheme. Finally, a total of 640 samples (i.e., 64 movement locations with 10 samples per movement, five measured and five generated samples) of the target movements were recorded during the data collection and preprocessing phases. Using five-fold cross-validation where data records per label were split into 80% (eight samples) for training, and 20% (two samples) for testing, there are 512 samples available for training and 128 samples available for testing. The evaluation phase uses additional hands-on datasets of 320 samples to simulate the real situation.

### 4.2. Hyperparameters of The Training Procedure

For the training algorithm of the ANN, categorical cross-entropy and Adam are used for the loss function optimization, respectively. The PIRILS expects labels to be provided in a one-hot representation [11]. The hyperparameters of $\beta_{1}$, $\beta_{2}$, ε, and the learning rate of Adam are default to 0.9, 0.99, $10^{-7}$, and 0.001, respectively [12]. The batch size is 4 and 50 epochs are used for training.

### 4.3. Stability

**Figure 7:**
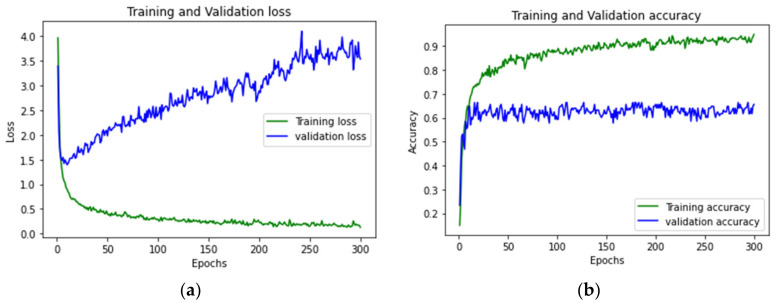
Loss and accuracy with varying the number of epochs from the ANN model with the SS method: (**a**,**b**) show the loss and accuracy of the LSTM, respectively.

This set of experiments evaluates the stability of the PIRILS. In Section 3.2, Figure 3 shows that the SS method has an influence on the performance of the ANN model. As shown in Figure 7, the loss and the accuracy of the training state are tracked using the SS method. After 50 epochs, the loss drops dramatically. Compared with the performance without applying the SS method, the performance metrics (e.g., the accuracy rates) of the LSTM model, when applying the SS model, can be improved by about 46%.

### 4.4. Reliability

This set of experiments characterizes the reliability of the ANN model with additional hands-on datasets of 320 samples. The classification of the ANN model is divided into five parts when there are two targets: $T_{initial}^{statistic}$, $\sigma_{initial}^{statistic}$, $T_{permutation}^{statistic}$, $\sigma_{permutation}^{statistic}$, and misclassification (false). Let T be the reliability measure given the target’s estimated position and let σ be the reliability measure with the positioning error of the specified classification category. Accordingly, referring to Equations (7) and (8) about $\Omega_{initial}^{score^{i}}$, $T_{initial}^{statistic}$ represents the reliability measure in the initial target order. Considering the cases of Items 1 and 2 in Section 3.4, $\sigma_{initial}^{statistic}$ represents the reliability measure of the positioning estimation error in the initial target order. Similarly, Equations (5) and (6) about $\Omega_{permutation}^{score^{i}}$, $T_{permutation}^{statistic}$ represent the reliability measure in the switched target order. As described in the cases of Items 4 and 5 in Section 3.4, $\sigma_{permutation}^{statistic}$ represents the reliability measure of the positioning estimation error in the switched target order. 

Applying the weights described in Algorithm 1 (i.e.,$\;score_{true}$ and $score_{deviation}$), the target index i, and the permutation with respect to the adjacent neighboring cell index j, the overall reliability of the classification,$\;R_{c}^{j},$ for the case of 2-target detection is the weighted sum of the four corresponding reliability measures $T_{initial}^{statistic}$, $\sigma_{initial}^{statistic}$, $T_{permutation}^{statistic}$, and $\sigma_{permutation}^{statistic}$, which yields:

(9) $$\begin{array}{cc} R_{c}^{j}=\sum_{i=1}^{2}\left(A+B\right) & \end{array}\;$$

with

$$A\;=\;score_{true}^{i}\cdot T_{initial\;\left(i\right)}^{statistic}+score_{true}^{j}\cdot T_{permutation\;\left(i\right)}^{statistic}\;$$

and

$$B\;=\;score_{deviation}^{i}\cdot \sigma_{initial\;\left(i\right)}^{statistic}+score_{deviation}^{j}\cdot \sigma_{permutation\left(i\right)}^{statistic}\;$$

**Figure 8:**
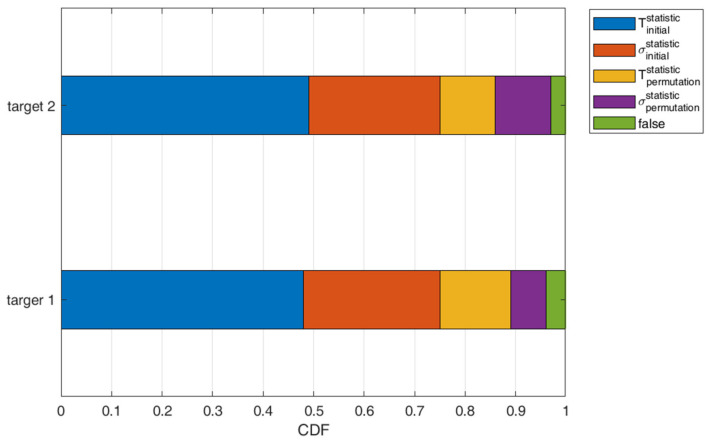
The normalized statistics for reliability measures using the validation dataset.

As shown in Figure 8, the statistics are normalized when the model is applied to the validation dataset. Consequently, the system reliability measure is 0.79, where $T_{initial}^{statistic}$, $\sigma_{initial}^{statistic}$, $T_{permutation}^{statistic}$, $\sigma_{permutation}^{statistic}$ are 0.49, 0.13, 0.13, and 0.04, respectively.

### 4.5. Localization Error

**Figure 9:**
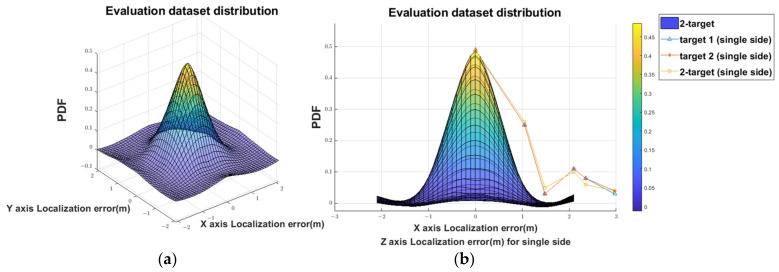
The pdf of the localization error: (**a**) 3D view and (**b**) X–Z view.

Figure 9 demonstrates the probability density function (PDF) of the prediction error of the localization in the 2-target detection. The results show that the errors of the ANN model approximately obey the zero-mean Gaussian distributions with a standard deviation of 1.03 m. The average system errors are shown in Table 3.

**Figure 10:**
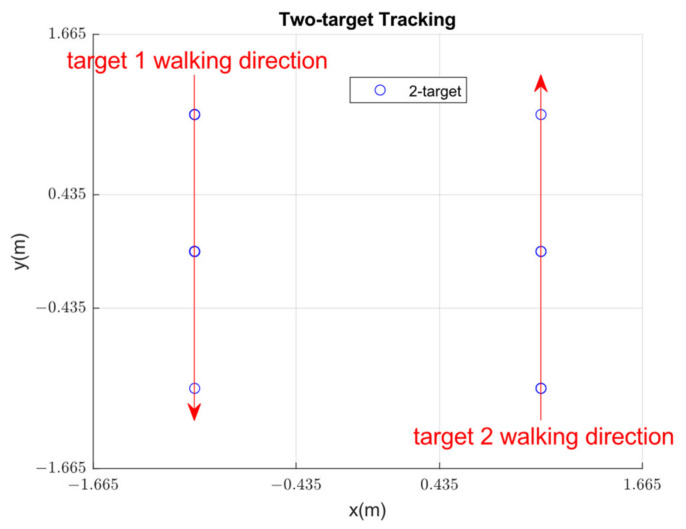
Two-target tracking with subjects walking in opposite directions.

Compared with the accuracy performance of the LSTM model without the SS method (average accuracy of 0.44), the performance of the LSTM model with the SS model (average accuracy of 0.64) is significantly improved. For multi-target scenarios, the PIRILS quantizes the analog PIR signals within a time frame and uses the SS method to improve the data diversity of the training dataset. Figure 10 shows a typical run of two-target tracking with subjects walking in opposite directions. Thus, the PIRILS provides a general solution to the two-target detection and localization problem and demonstrates the system’s feasibility for multi-target tracking and localization.

## 5. Discussion

To assess the system performance and support the accuracy information, this section explores the sensor characteristics, deployment density, model properties, response time, and deployment scenarios. 

### 5.1. Sensor Characteristics

This subsection summarizes the PIR sensors and modules adopted in the existing works. Studies in [2,5,13] adopt the PIR sensor Tranesen-PCD-2F21, and the Fresnel lens array YUYING-8719. The PIR sensor, along with the Fresnel lens array, is put on a rotation plate which can rotate at a pre-defined constant speed of $15^{{}^{\circ}}/s$. Yang et al. [14] select PIR C172 and PIR Cone Optics TR230 by KUBE. Compared with the conventional Fresnel lens, TR230 is much smaller and can be deployed on a large scale. You [6] employs IRA-E710 from Murata Manufacturing Company, whose both vertical and horizontal FOVs are 90°. Four PIR sensors are separated by 2-cm space so that large multizone Fresnel lenses could be conveniently attached or detached for testing. Instead of using multizone Fresnel lenses, single-zone Fresnel lenses, IML-0637 from Murata Manufacturing Company, are employed for analyzing output signals. Lu et al. [7] utilize the multizone Fresnel lens of the PIR module (HC-SR510) with two-layer coded masks. Each sensor node contains two PIR sensors, where Fresnel lens arrays and masks are used to change the FOV of each sensor. The PIR sensors are deployed on the walls to measure human presence. In this work, similar to [7], a multizone Fresnel lens of the PIR module (HC-SR510) and a reference structure are applied to divide the specific detection area.

### 5.2. Performance Comparison 

**Figure 11:**
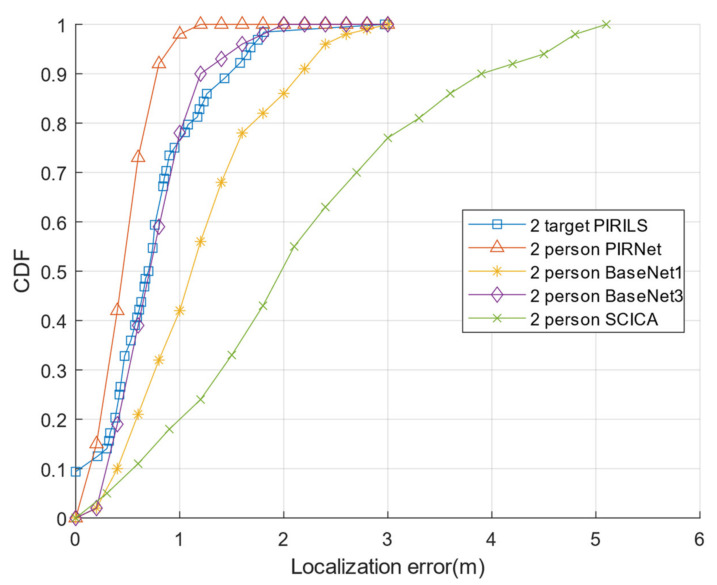
Comparison of the CDFs of the absolute localization error.

Most existing works with a ceiling-deployed strategy (e.g., [15,16,17,18]) focus on single-target tracking and localization. One recent work focuses on detecting the movement directions of multiple targets, though not for tracking and localization [6]. Figure 11 compares the cumulative distribution functions (CDFs) of the absolute localization errors of the PIRILS with existing PIR-based methods from [1,19] (e.g., PIRNet, BaseNet1, BaseNet3, and the SCICA-based method) in 2-target localization with a wall-deployed strategy. The average absolute localization errors of the PIRILS, PIRNet, BaseNet1, BaseNet3, and SCICA-based methods are respectively 0.67 m, 0.46 m, 1.20 m, 0.76 m, and 1.64 m. Observe that the localization error of the method based on SCICA is much higher compared with the existing methods. We echo the result of Yang et al. [1] that the FOVs corresponding to different targets have overlapping spectra. Davies argues that the SCICA method handles independent signals poorly due to the substantial overlap of their spectra [19]. Moreover, Figure 9 shows that the PIRILS has better positioning performance in the initial state and a similar characteristic curve when compared with other methods. Note that, due to the height of the ceiling, the detection range of a PIR sensor may be underutilized; thus, a ceiling-mounted system may receive less information than a wall-mounted system. While a ceiling-deployed system does not have an advantage in precision, it does have an advantage in practicality.

Table 4 compares the average localization error and the deployment density of the PIRILS with other PIR-based methods. In the PIRILS, the performance of the localization error is affected by the spatial resolution. The deployment density in the PIRILS is limited by the sensor locations on the ceiling. In our previous research [4], the maximum detection range was 5 ~ 7 m, so the minimum deployment density was 0.08 ~ 0.04 sensors/$m^{2}$, which is similar to existing methods.

To further explore the system characteristics, the response times of tracking operations using different methods are examined, considering the time consumptions of data collection, data preprocessing, and model operation. The system in [1] performs the tracking task in about 3.23 s, including the time required for data collection and data preprocessing (3 s) and model operation (0.23 s). As reported in [13], the response time of a tracking operation is about 5.02 s, including the time for data collection (5 s of data) and model operation (20 ms). For the system in [7], the running time for distributed message passing is about 1.8 s. Regarding the procedures of data collection, the preprocessing modules of sensor selection and calibration, and the message passing algorithm, a longer delay will be expected in the tracking task (>1.8 s). For the proposed PIRILS system, the response time of a tracking operation is about 5.32 s, including the time needed to collect data (5 s), data preprocessing (0.31 s), and model operation (12 ms). Referring to the above-mentioned experimental results, it is suggested that the time consumption of data collection and data preprocessing is the dominant term in the response time of tracking operations.

## 6. Conclusions

This paper proposes the PIRILS, a non-wearable system for cooperative indoor human localization that utilizes a data augmentation strategy and a lightweight system with deep learning to achieve PIR-based multi-target positioning and labeling. The results show that the PIRILS increases data diversity and positioning accuracy while achieving system performance competitive with other state-of-the-art systems. In the future, in addition to considering a fixed number of targets (as do most multi-target PIR methods, e.g., [2,7,13]), we plan to extend the PIRILS strategy to allow tracking of a changing number of targets. This can be accomplished by data preprocessing and running identical algorithms (e.g., assuming n, n + 1, and n − 1 targets) in order to select the best-fitting hypothesis and dynamically estimate the target number, to adaptively control the window size of collected data, and to further improve the ANN model structure for multi-target tracking and localization.

## Figures and Tables

**Table 1:** Summary of the PIR Detection Systems.

Reference	Sensor Location	Processing Techniques	Observation Space	Average RMSE (m)
Sensor Selection and Calibration Method [7]	Wall	Probability Model-based Calibration	6 m × 6 m	0.35
PIRNet [1]	Wall	Modular learning	7 m × 7 m	0.46
DeepPIRATES [5]	Wall	Non-end-to-end learning	7 m × 7 m	0.73
[6] with CNN	Ceiling	Deep learning/Classifier	1.9 m × 1.9 m	90% accuracy rate
PIRILS	Ceiling	Deep learning/Classifier	3.3 m × 3.3 m	0.67

**Table 2:** The situation of $Cell\left(c\right)$ covered by the detection areas of the PIR modules in a detector.

$Cell\left(c\right)$	PIR Module of a Detector			
	1st	2nd	3rd	4th
1	1	0	0	0
2	1	0	1	0
3	0	0	1	0
4	1	1	0	0
5	1	1	1	1
6	0	0	1	1
7	0	1	0	0
8	0	1	0	1
9	0	0	0	1

**Table 3:** The average MAE of 2-target localization.

Experimental Route	$\mathrm{Avg}.\;MAE_{x}$	$\mathrm{Avg}.\;MAE_{y}$	Avg. MAE (m)
Target 1	0.52	0.43	0.67
Target 2	0.43	0.52	0.67
2-target Tracking	0.48	0.48	0.68

**Table 4:** Comparison of PIR-based device-free localization methods with two targets.

Method	PIRILS	PIRNet [1]	[5]	[13]	[14]	[7]	BaseNet1 [1]	BaseNet3 [1]	SCICA [19]
Mean Error (m)	0.68	0.46	0.73	0.87	0.50	0.47	1.20	0.76	1.64
Deployment Density (sensors/$m^{2}$)	0.36	0.08	0.08	0.08	0.34	0.89	0.08	0.08	0.08
Require Training Data	Yes	Yes	Yes	No	No	Yes	Yes	Yes	Yes
Require Fine-Tuning	Yes	Yes	No	No	No	Yes	Yes	Yes	Yes
Response Time (s)	5.32	3.23	N/A	5.02	N/A	>1.8	N/A	N/A	N/A
Sensor Location	Ceiling	Wall	Wall	Wall	Floor	Wall	Wall	Wall	Wall

## Data Availability

The data presented in this study are available on request from the corresponding author.
